# Exacerbations and Management of Asthma in Viral Lower Respiratory Tract Infections: The Significance of Immunoglobulin E

**DOI:** 10.1002/iid3.70386

**Published:** 2026-02-25

**Authors:** Mandana Akhavan, Parastoo Yousefi, Alireza Tabibzadeh

**Affiliations:** ^1^ Cell and Molecular Biology, Department of Biology, Science and Research Branch Islamic Azad University Tehran Iran; ^2^ Division of Molecular Biology, Medical Mycology and Parasitology Department, School of Public Health Research Center Tehran University of Medical Sciences Tehran Iran; ^3^ Department of Virology, School of Medicine Iran University of Medical Sciences Tehran Iran; ^4^ Rajaei Clinical Research Development Alborz University of Medical Sciences Karaj Iran

**Keywords:** asthma, COVID‐19, IgE, influenza, lower respiratory tract infections (LRTI), RSV

## Abstract

**Background:**

Viral‐respiratory infections are the most prevalent illness among humans. A viral infection affecting lower respiratory tract infections (LRTI) is a critical health concern worldwide. The COVID‐19 pandemic has significantly impacted respiratory health, particularly in individuals with asthma. Other viral respiratory infections and asthma are critical concerns, either. The current study aimed to discuss how elevated IgE levels can influence viral LRTI and potentially exacerbate asthma symptoms, as well as biological treatments targeting IgE in managing asthma.

**Materials and Methods:**

The search was conducted in electronical databases (including PubMed, Scopus, Google Scholar, and so on). all obtained documents were listed and reviewed by two independent authors. All relevant studies were included and used for final assessment and data collection.

**Results:**

IgE is a crucial mediator in the pathophysiology of asthma, particularly in type 2‐high (T2‐high) asthma, where it drives allergic responses and airway inflammation. The interaction between COVID‐19 and asthma has illustrated that asthmatic patients may experience increased respiratory symptoms following COVID‐19 infection. Interestingly, T2‐high asthmatics may have had some protection against severe COVID‐19 outcomes, highlighting the need for a nuanced understanding of asthma management during and after the pandemic.

**Conclusion:**

Viral infections, particularly those caused by human rhinoviruses, are a significant trigger for asthma exacerbations. These infections can lead to heightened serum IgE levels, which play a vital role in the immune response and the worsening of asthma symptoms. The Th2 inflammatory pathway is frequently upregulated during these infections, associated with increased production of cytokines such as IL‐4, IL‐5, and IL‐13, which aggravate asthma symptoms. Additionally, viral infections can compromise the airway epithelium, resulting in greater exposure to allergens and irritants, and disrupt the balance between Th1 and Th2 cytokines, leading to more severe exacerbations.

**Abbreviations**
ACE2: angiotensin‐converting enzyme 2
AHR: airway hyperresponsiveness
ARDS: acute respiratory distress syndrome
COPD: chronic obstructive pulmonary disease
COVID‐19: Coronavirus disease 2019
DCs: dendritic cells
HRV: human rhinovirus
ICS: inhaled corticosteroids
IFN‐γ: interferon‐gamma
IgE: immunoglobulin E
LRTI: lower respiratory tract infections
PRRs: pattern recognition receptors
RSV: respiratory syncytial virus
T2‐high: type 2‐high
T2‐high: type 2‐high
T2‐low: type 2‐low
Th: T helper
TNF: tumor necrosis factor

## 1 Introduction

Lower respiratory tract infections (LRTIs) represent a significant global health concern, particularly affecting children and the elderly. In 2015, there were approximately 291 million cases of LRTIs, leading to 2.74 million deaths; making it one of the leading causes of mortality from communicable diseases, second only to Coronavirus disease 2019 (COVID‐19) in recent years [1]. LRTIs are most prevalent in children under five, with an incidence rate of approximately 12,197.8 cases per 100,000. Pneumonia, the most common type of LRTI, is responsible for over 61% of hospitalizations in this age group. The impact of LRTIs is especially pronounced in low‐ and middle‐income countries, where the pneumonia mortality rate for young children was 12.5 per 100,000 in 2019 [2]. Recent estimates in 2021 indicate that there were around 344 million incident episodes of lower respiratory infections globally, with approximately 2.18 million deaths attributed to these conditions [3].

Recent studies have well‐documented the association between LRTIs in early childhood and the subsequent development of asthma. Specifically, viral LRTIs occurring in children under the age of two are linked to a significantly increased risk of developing asthma later in life, with an odds ratio of 5.0 (95% CI: 3.3–7.5) indicating a strong correlation that persists up to 20 years of age. This relationship is particularly pronounced in cases requiring hospitalization, where the odds ratio can rise as high as 14.2 for developing asthma compared to controls without LRTIs [4]. Furthermore, the severity of the initial infection plays a critical role; children who experience severe LRTIs are at a heightened risk for asthma, emphasizing the need for early intervention and monitoring in pediatric populations [5, 6]. These findings suggest that healthcare providers should be vigilant about the long‐term respiratory health of children who have experienced viral LRTIs.

The burden of viral lower respiratory infections extends beyond direct health impacts; they also strain healthcare resources significantly. For example, respiratory syncytial virus (RSV) alone leads to substantial healthcare utilization, including emergency visits and hospitalizations during peak seasons. The unpredictability of RSV activity, especially post‐COVID‐19, has resulted in increased susceptibility among populations previously exposed to the virus [7, 8]. Both influenza and RSV are critical contributors to the global burden of lower respiratory infections. Their impact on public health necessitates ongoing surveillance, vaccination efforts, and targeted interventions to reduce morbidity and mortality associated with these viral pathogens. Addressing these infections is essential for improving health outcomes in affected populations worldwide.

The COVID‐19 pandemic has significantly impacted individuals with asthma, raising important questions about the interplay between asthma management and the role of Immunoglobulin E (IgE). Asthma, a chronic respiratory condition characterized by airway inflammation and hyperreactivity, is predominantly mediated by type 2 immune responses involving T helper (Th) 2 cells, which produce key cytokines such as interleukin 4 (IL‐4) and IL‐13. These cytokines are crucial for producing allergen‐specific IgE and accumulating eosinophils in the airways, contributing to asthma pathophysiology [9].

Interestingly, research indicates that individuals with allergic asthma may experience a lower susceptibility to severe COVID‐19 outcomes. This phenomenon may be attributed to various factors, including the anti‐inflammatory effects of inhaled corticosteroids (ICS), commonly prescribed for asthma management. ICS has been associated with reduced expression of angiotensin‐converting enzyme 2 (ACE2), the receptor utilized by severe acute respiratory syndrome coronavirus 2 (SARS‑CoV‑2) for cellular entry, thereby potentially lowering the risk of severe infection [9, 10]. However, the relationship between asthma and COVID‐19 remains complex, with studies showing contradictory results regarding the risk of infection and severity among asthmatic patients [11].

Viral LRTIs, such as those caused by influenza and RSV, elicit complex immune responses involving both innate and adaptive immunity. The initial defense is mounted by the innate immune system, which includes alveolar macrophages and dendritic cells (DCs) that recognize viral components through pattern recognition receptors (PRRs). These cells rapidly respond to infection by producing pro‐inflammatory cytokines and recruiting additional immune cells, including neutrophils and monocytes, to the site of infection [12, 13]. As the disease progresses, adaptive immunity is activated, characterized by the proliferation of virus‐specific T cells and B cells, producing antibodies and cytotoxic T lymphocytes that target infected cells [14]. However, this immune response can become dysregulated, potentially resulting in excessive inflammation and tissue damage, which may contribute to severe disease outcomes such as pneumonia or acute respiratory distress syndrome (ARDS) [15].

Moreover, elevated IgE levels have been linked to exacerbated responses to viral infections, suggesting that IgE plays a significant role in the clinical outcomes of asthmatic patients during the pandemic [16]. This manuscript aimed to discuss how elevated IgE levels can influence viral infections and potentially exacerbate asthma symptoms and also biological treatments targeting IgE in managing asthma.

## 2 Asthma Pathophysiology and Ige

Th2 activation and cytokines are crucial for producing allergen‐specific IgE and accumulating eosinophils in the airways, contributing to asthma pathophysiology [9]. Th2 cells from asthmatics patients represents a distinct gene expression signature including upregulation of interleukin 17 receptor B (IL‐17RB) or IL‐25 receptor, Carnitine Palmitoyl transferase 1 A (CPT1A) and Caspase 2 (CASP2) which all are important in Th2 responses, and downregulation on Dual Specificity Phosphatase 10 (DUSP10), Zinc Finger And BTB Domain Containing 10 (ZBTB10), and GABA Type A Receptor Associated Protein Like 1 (GABARAPL1) which inhibits T cell activation and survival by c‐Jun N‐terminal kinase (JNK) signaling pathway and promoting autophagy flux [17].

Asthma can be classified into different phenotypes based on underlying mechanisms and inflammatory profiles. A key distinction is between type 2‐high (T2‐high) and type 2‐low (T2‐low) asthma. T2‐high asthma is characterized by elevated levels of Th2 cytokines (IL‐4, IL‐5, IL‐13) and is often associated with atopy and elevated IgE levels. In this phenotype, eosinophils play a crucial role; they are recruited to the airways in response to Th2 cytokines, leading to further inflammation and tissue damage [18, 19]. In contrast, T2‐low asthma is characterized by a different inflammatory profile that may involve neutrophilic inflammation without significant involvement of IgE or eosinophils. This phenotype may respond differently to treatment modalities compared to T2‐high asthma, highlighting the importance of understanding these distinctions for effective management strategies [20, 21]. The differential roles of eosinophils and cytokines in T2‐high asthma underscore the complexity of asthma as a heterogeneous disease requiring tailored therapeutic approaches.

IgE plays a pivotal role in the pathophysiology of asthma, particularly in allergic asthma. The mechanism of IgE‐mediated allergic responses includes the sensitization phase, the production of allergen‐specific IgE by B cells, the IgE attachment to FcεRI on effector cells, and the release of pro‐inflammatory mediators. These mediators contribute to both acute and chronic symptoms of asthma, such as bronchoconstriction, airway inflammation, and increased mucus production [21, 22, 23]. The impact of IgE on airway hyperreactivity and inflammation is significant. Elevated levels of serum IgE are correlated with increased severity of asthma and bronchial hyperresponsiveness. Furthermore, IgE not only facilitates immediate hypersensitivity reactions but also contributes to chronic inflammation through its effects on various immune cells, including eosinophils and Th2 cells [20, 21, 22].

The interplay between IgE, mast cells, eosinophils, and Th2 cells is central to the pathophysiology of asthma and significantly affects asthma symptoms. The Th2 cells produce key cytokines such as IL‐4, IL‐5, and IL‐13. These cytokines are crucial for promoting IgE class switching in B cells, resulting in the production of allergen‐specific IgE antibodies [24, 25]. In this regard, IL‐5 can augment the maturation and survival of eosinophils in the lungs [26, 27]. IL‐4 is vital for the differentiation of naïve T cells into Th2 cells and facilitates IgE class switching in B cells [28, 29]. IL‐13 induces mucus production due to goblet cell hyperplasia and airway remodeling. The signaling pathways activated by IL‐13, particularly through the IL‐13Rα1 receptor, are crucial for airway hyperresponsiveness (AHR). Both IL‐4 and IL‐13 share a common receptor component (IL‐4Rα), allowing them to activate similar signaling pathways, notably the signal transducer and activator of transcription 6 (STAT6) pathway. This shared mechanism highlights their collaborative role in promoting airway inflammation and remodeling. However, while IL‐4 is more involved in initiating allergic responses and regulating IgE production, IL‐13 plays a more direct role in the structural changes and hyperreactivity associated with asthma. However, IL‐4 and IL‐13 contribute to asthma pathogenesis through their distinct yet complementary roles in facilitating Th2 differentiation, eosinophilic inflammation, mucus production, airway remodeling, and hyperresponsiveness. Targeting these cytokines or their signaling pathways offers a promising therapeutic approach for managing asthma [30, 31, 32]. The interplay between IgE, mast cells, eosinophils, and Th2 cells creates a complex network that drives the inflammatory processes in asthma. This network not only contributes to immediate allergic responses but also plays a significant role in chronic inflammation and airway remodeling, ultimately affecting the severity and frequency of asthma symptoms.

## 3 COVID‐19 and Its Effects on Asthma

### 3.1 Severe Phase of COVID‐19 and Asthma

COVID‐19 has significantly impacted individuals with asthma, leading to both exacerbations of symptoms and challenges in management. A study found that among asthmatic patients who contracted COVID‐19, approximately 33.9% experienced a worsening of their asthma control post‐infection, necessitating increased medication to manage their symptoms effectively [33]. While some studies indicate that asthma patients may be less susceptible to severe COVID‐19 outcomes, the persistent effects on asthma control highlight the need for tailored management strategies for this population [10, 34].

COVID‐19 has profound respiratory implications, particularly for individuals with pre‐existing conditions like asthma. The virus primarily affects the respiratory tract, causing inflammation and damage to lung tissues, which can exacerbate asthma symptoms and lead to new‐onset asthma in previously healthy individuals. Studies indicate that COVID‐19 may significantly increase the risk of developing respiratory diseases, including asthma, especially in patients who experienced moderate to severe symptoms during their infection. For instance, a study highlighted that reinfection with COVID‐19 correlates with a heightened risk of respiratory diseases, including asthma and chronic obstructive pulmonary disease (COPD) [35]. COVID‐19 primarily affects the respiratory system, leading to inflammation and damage to lung tissues. This can exacerbate existing asthma symptoms and complicate management strategies for individuals with the condition. Research indicates that patients with asthma and COVID‐19 may experience more severe respiratory distress, which can lead to longer‐term complications in lung function [36].

### 3.2 Long Covid and Asthma

Long COVID presents additional challenges, as individuals may experience persistent respiratory symptoms such as breathlessness and cough even after recovery from the acute phase of the ailment. These symptoms often require ongoing management and rehabilitation [37, 38]. Furthermore, many patients reported overlapping respiratory symptoms from both conditions, complicating their overall health management. In another analysis, 73.7% of patients with long‐term COVID‐19 reported worse breathing compared to pre‐infection levels, highlighting the persistent effects on asthma control and the need for tailored management strategies for this population [39].

The interplay between IgE levels, asthma control, and COVID‐19 highlights the need for personalized management strategies. Patients with asthma should be closely monitored for respiratory symptoms during viral infections, and treatment plans may need to be adjusted based on their IgE levels and overall asthma control. The use of ICS has been suggested to mitigate severe inflammatory responses associated with COVID‐19, further complicating treatment approaches [9, 40].

### 3.3 A Triangle of COVID‐19, Ige and Asthma

IgE is a key player in the pathophysiology of asthma, particularly in type 2 inflammation, which is characterized by elevated levels of IgE and eosinophils. In asthmatic patients, IgE contributes to airway hyperreactivity and inflammation, leading to exacerbations during respiratory infections. Studies have shown that the presence of IgE can weaken the interferon‐mediated antiviral response, which is crucial for controlling viral infections like SARS‐CoV‐2. This weakened response may increase the risk of severe outcomes in asthmatic patients infected with COVID‐19. However, the anti‐IgE biologic treatment omalizumab has been shown to reduce asthma exacerbations triggered by respiratory viruses, suggesting a potential protective effect against severe COVID‐19 outcomes in some patients with asthma [41].

Interestingly, research indicates that certain asthma endotypes may confer a degree of protection against COVID‐19. For instance, allergic asthma (characterized by high levels of IgE) has been associated with lower expression of ACE2 2, the receptor that SARS‐CoV‐2 uses to enter cells. Lower ACE2 expression in airway epithelial cells may reduce the susceptibility to infection. Conversely, non‐allergic asthma may not exhibit this protective mechanism, highlighting the importance of understanding individual patient profiles when assessing risk [41, 42].

The immune response in asthmatic patients can also influence their reaction to COVID‐19. Type 2 cytokines such as IL‐4 and IL‐13 reduce ACE2 expression, potentially providing a protective effect against SARS‐CoV‐2 infection. However, other cytokines like IL‐17 can increase ACE2 levels, complicating the immune landscape in asthmatic individuals [9, 43]. Additionally, eosinophils, typically elevated in allergic asthma, may play a dual role; while they are often associated with inflammation, they can also contribute to antiviral defenses in the lungs during viral infections [9, 42].

During COVID‐19 infection and immune responses, cytokines play a critical role. Previous studies have reported a dramatic increase in IL‐25 levels in COVID‐19 patients [44]. IL‐25 (known as IL‐17E) could be considered a double‐edged sword in infectious diseases. It can activate type 2 immunity via Th2 cytokines, which leads to asthma exacerbation or development, and also provokes group 2 innate lymphoid cells (ILC2), augmentation of Th2 differentiation, which could be a critical step in immune responses and pathogen clearance [45]. The CPT1A expression level during COVID‐19 seems to be increased [46]. This finding can highlight the augmentation of asthma in COVID‐19 based on the asthmatic patients' Th2 expression profile. As we mentioned earlier, Th2 cells from asthmatic patients represent a distinct gene expression signature including upregulation of IL‐25 receptor and CPT1A, which can promote Th2 survival and responses through JNK signaling and autophagy [17]. In this regard, a considerable crosslink between COVID‐19 and the JNK signaling pathway was reported previously. The JNK signaling pathway plays an important role during coronavirus replication [47]. Also, COVID‐19 infection could be an augmentation factor for the JNK signaling pathway, which seems to be a critical factor during Th2 activation [48].

## 4 Influenza Infection and Asthma

### 4.1 Influenza Virus Virological and Epidemiological Features

Another important LRTI viral agent is Influenza. Seasonal influenza is responsible for approximately 3 to 5 million cases of severe illness and between 290,000 to 650,000 respiratory deaths worldwide each year. The virus predominantly affects children and the elderly, with hospitalization rates significantly higher in these groups during peak seasons [3, 49].

Influenza is due to three virus types: A, B, and C. The 20th century saw three major pandemics: the 1918 Spanish flu, affecting over 100 million people; the 1958 Asian flu; and the 1968 Hong Kong flu, each causing over a million deaths. A key finding in the lungs of those who succumb to the disease is necrotizing bronchiolitis, characterized by cell death in bronchiolar walls, tissue swelling, and a mix of inflammatory cells infiltrating the affected areas [50]. Influenza infection significantly impacts individuals with asthma, leading to increased morbidity and exacerbations of asthma symptoms. The influenza virus can exacerbate airway inflammation, causing complications such as pneumonia, which poses a greater risk for asthmatics than the general population. Studies have shown that during the 2009 H1N1 pandemic, asthma was a common comorbidity among hospitalized patients, with approximately 25% of those affected being asthmatics. Interestingly, these patients exhibited lower mortality rates and less need for intensive care compared to non‐asthmatics, suggesting a complex interaction between asthma and influenza infection [51, 52].

### 4.2 Influenza Virus and Ige in Asthma

Influenza infection has been linked to the exacerbation of asthma symptoms, particularly through its influence on IgE production. Research indicates that respiratory viral infections, including influenza A, can enhance sensitization to inhaled allergens, which may lead to increased airway hyperresponsiveness and asthma exacerbations. In a study involving mice, it was found that influenza A virus infection significantly increased the levels of ovalbumin‐specific IgE when combined with ovalbumin sensitization. This suggests that the presence of the virus alters the immune response, promoting IgE production in response to environmental antigens that would typically not elicit such a strong reaction in uninfected individuals [53]. The underlying mechanisms are thought to involve changes in T cell populations and cytokine profiles, particularly an increase in CD8 + T cells and cytokines like IL‐5, which is known to upregulate IgE synthesis [51, 53]. The primary immune effector mechanism in experimental influenza is T‐cell–mediated cytotoxicity that kills infected epithelial cells, bronchial lining cells, and Type‐II pneumocytes, like viral exanthema. The bronchial lesions of the experimental model of asthma in mice are preceded by an immune complex vasculitis and not an IgE‐mediated mast cell mechanism [50]. The mechanism through which CD8 + T cells enhance IgE production involves their ability to secrete cytokines such as IL‐5, which promotes eosinophilic inflammation and B cell class switching to IgE. Additionally, virus‐infected CD8 + T cells may co‐express markers that facilitate interactions with other immune cells, amplifying the Th2‐type response and increasing allergen‐specific IgE levels. This response not only exacerbates asthma symptoms but also increases susceptibility to further allergen exposure, creating a feedback loop that worsens airway inflammation and hyperresponsiveness [53, 54]. Moreover, CD8 + T cells are essential for clearing influenza virus infections by inducing apoptosis in infected cells and producing antiviral cytokines like Interferon gamma (IFN‐γ) and Tumor necrosis factor (TNF). However, their dual role in promoting both protective immunity and immune pathology highlights a delicate balance. While these cells are indispensable for controlling viral replication, their involvement in IgE‐mediated responses during influenza infection can lead to detrimental outcomes in individuals with pre‐existing allergic conditions such as asthma [53, 55].

## 5 RSV and Asthma

### 5.1 RSV Virological and Epidemiological Features

RSV is a leading cause of LRTIs in children under 2 years of age. It infects nearly 90% of children by their second birthday, with about 33 million cases of RSV‐associated lower respiratory tract illnesses estimated annually. This results in approximately 3 million hospitalizations and up to 199,000 childhood deaths globally, particularly impacting resource‐limited countries [56, 57]. Among infants with RSV, about 40% develop bronchiolitis, a severe form of lower respiratory illness [56].

### 5.2 RSV and IgE in Asthma

RSV infection triggers a robust immune response characterized by the production of IgE antibodies. Studies have shown that infants with positive RSV‐specific IgE at the age of one are nearly six times more likely to develop asthma compared to those without such antibodies [58]. This suggests that RSV may induce an atopic response, promoting the production of IgE through Th2 immune pathways. The Th2 response is typically associated with allergic reactions and asthma, characterized by elevated levels of cytokines such as IL‐4, IL‐5, and IL‐13, which further stimulate IgE production and eosinophilic inflammation in the airways [59, 60]. The mechanisms underlying the association between early RSV infection and subsequent asthma development are multifaceted. One theory posits that RSV may enhance sensitization to environmental allergens, thereby promoting atopic responses. This occurs through the virus's Th2‐trophic effects, which can lead to an imbalance favoring Th2‐mediated immune memory against inhalant allergens [59]. Additionally, RSV infection has been shown to induce the release of pro‐inflammatory cytokines from airway epithelial cells, which can recruit immune cells and exacerbate airway inflammation [60, 61]. Furthermore, research indicates that children who experience severe RSV infections requiring hospitalization are at a heightened risk for allergic sensitization and asthma later in life [58, 59].

IgE acts as a critical mediator in allergic responses, initiating an inflammatory cascade upon allergen exposure. This process releases pro‐inflammatory mediators, contributing to both acute and chronic asthma symptoms [62]. Elevated serum IgE levels are associated with an increased risk of developing asthma, although the correlation between total IgE levels and asthma severity is less clear [21]. Understanding the role of IgE in the context of RSV infection has important clinical implications. For instance, targeted therapies such as anti‐IgE treatments could potentially mitigate the long‐term effects of RSV on respiratory health by reducing IgE‐mediated inflammation and airway hyperreactivity [25, 61].

## 6 Other Viral Infections and Asthma

Studies indicate that co‐infections with other viruses are common. For instance, human rhinovirus (HRV) and adenovirus often co‐occur with influenza and RSV, complicating clinical outcomes [49, 63]. Asthma is characterized by chronic airway inflammation and hyperresponsiveness, which viral infections like influenza can aggravate. Viral infections, particularly in early life, are linked to the development and exacerbation of asthma; for instance, RSV and rhinovirus infections are known to increase the risk of subsequent asthma episodes [25, 64]. Another study emphasized that viral infections are prevalent triggers for acute asthma attacks, underscoring the need for effective vaccination strategies tailored for individuals with asthma to mitigate these risks [65].

The interplay between viral infections and asthma is complex. Viral infections can trigger asthmatic responses through various immunological pathways. For instance, viral infections may alter the immune response in individuals with asthma by shifting cytokine profiles from protective Th1 responses to more inflammatory Th2 responses associated with severe asthma exacerbations [66]. Moreover, patients with asthma often exhibit a delayed antiviral response due to impaired type I interferon production, leaving them more susceptible to viral replication and exacerbated symptoms [66, 67].

The relationship between viral infections and asthma is further complicated by the role of IgE in allergic responses. Higher levels of allergen‐specific IgE are associated with an increased risk of asthma exacerbations following viral infections. For instance, rhinovirus infections have been shown to correlate with more severe asthma symptoms, especially in children with elevated IgE levels. This connection highlights a synergistic effect where viral infections not only trigger asthma symptoms but also exacerbate underlying allergic conditions by promoting IgE‐mediated responses [68, 69]. Viral infections, especially in children, are a leading cause of respiratory illnesses, contributing to complications. Key viruses include RSV, influenza, rhinovirus, and coronaviruses, which can lead to mild to severe infections. Due to their developing immune systems and exposure in group settings, children typically face six viral respiratory infections per year, presenting symptoms like nasal congestion and cough. Severe cases can lead to wheezing and cyanosis in infants [70].

Rhinoviruses are the most common cause of the common cold and are particularly notorious for exacerbating asthma symptoms. They have been linked to increased wheezing episodes and asthma attacks in both children and adults. Research indicates that early‐life rhinovirus infections may predispose individuals to develop atopic asthma later in life, likely due to allergic sensitization mechanisms. Specific IgE antibodies are associated with a greater risk of wheezing following rhinovirus infections, suggesting a synergistic relationship between viral infections and allergic responses [68].

## 7 Management Strategies for Asthma Patients

Over the years, the main treatment options for asthma have been immunomodulators such as corticosteroids and beta‐agonists. Short‐Acting Beta‐Agonists (SABAs) represent a rapid‐onset bronchodilator function that could be relieving for an acute asthma presentation. This made SABAs a treatment of choice for asthma for years. Due to recent research, SABA monotherapy overuse seems to be associated with increased rates of mortality and exacerbations in asthma. This finding suggests the SABA combination of ICS as a recommended treatment [71]. ICS, oral corticosteroid (OCS), and Systemic corticosteroids (SCS) are another classical option for asthma management. Recent findings suggest OCS are associated with comorbidities and mortality for long‐term treatment of asthma [72]. Also, studies recommend against ICS for asthma management for prevention of critical side effects, including osteoporosis and cardiovascular complications [73]. Regarding corticosteroid side effects, ICS remains a major treatment option in asthmatic patients due to reducing the risk of exacerbation and its great potential for personalization [74]. Since this therapeutic strategy is faced with a variety of side effects and limitations. In this regard, new approaches for more effective and targeted treatment, such as anti‐IgE biologics, were developed [75].

Management strategies for IgE‐mediated allergies include antihistamines for immediate relief and corticosteroids as controllers. Allergen‐specific immunotherapy (SIT) is also an effective long‐term treatment option. In severe cases, anti‐IgE biologics such as omalizumab have been employed to reduce IgE levels and mitigate allergic responses [43].

An anti‐IgE biologic for asthma is known as Omalizumab. Omalizumab was approved by the FDA in 2003 for the treatment of asthma in patients aged more than 12 years. Omalizumab is an IgG1κ humanized recombinant monoclonal antibody that binds to the Cε3 region of the Fc fragment of IgE at the site of FcϵR1 binding with acceptable effectiveness for the treatment of asthma. The Omalizumab reduces IgE level in serum and inhibits IgE binding to cellular receptors, which will reduce Th2 cytokines and prevent all the mentioned mechanisms of pathogenesis [76, 77]. Regarding the great efficacy of Omalizumab, other monoclonal antibodies were developed for controlling asthma based on the natural pathophysiology of the disease. Mepolizumab (anti‐IL‐5 antibody) and Benralizumab (anti‐IL‐5 receptor antibody) are IL‐5/IL‐5R antibodies that represent a promising future for asthma treatment [78, 79].

Another aspect of treatment will be focused on antiviral therapy. Antiviral therapies are critical for managing viral LRTIs caused by pathogens such as RSV, influenza, etc. Ribavirin, an antiviral agent, has been extensively studied for RSV infections. It can be administered via oral, intravenous, or aerosolized routes and has shown efficacy in reducing viral loads in immunocompromised patients. However, its clinical benefits remain controversial due to frequent treatment failures and side effects like hemolytic anemia [80, 81, 82]. In case of RSV, there are other therapeutic and prophylactic agents as well. Palivizumab, Nirsevimab, and Motavizumab (next generation of Palivizumab) are three anti‐RSV monoclonal antibodies, and RSV‐IVIG (is an intravenous polyclonal immunoglobulin against RSV) is approved as a clinically effective option for post‐exposure prophylaxis and treatment. Also, different FDA‐approved vaccine platforms for RSV are available. These vaccines include recombinant RSV glycoprotein vaccines for instance RSVPreF3 (Arexvy) and RSVpreF (Abrysvo), and mRNA technology vaccine, include mRNA‐1345 (mRESVIA) [83].

For influenza, neuraminidase inhibitors such as oseltamivir and zanamivir are widely used. These drugs inhibit the enzymatic activity of viral neuraminidase, effectively reducing the severity and duration of illness. Laninamivir, approved in Japan, offers a single‐dose inhalation alternative with comparable efficacy to oseltamivir for influenza A and B [84]. Additionally, investigational agents like viramidine (a ribavirin analog) have demonstrated anti‐influenza activity in vitro and animal studies [80]. Another anti‐influenza treatment that was approved in 2018 is Baloxavir Marboxil. Baloxavir Marboxil is a treatment option different from neuraminidase inhibitors and acts as a cap‐dependent endonuclease inhibitor. Different mechanism of action makes Baloxavir a treatment of choice for neuraminidase inhibitor‐resistant strains of influenza type A and B [85].

This path to the achievement of a clinically effective antiviral has accelerated in recent years, especially after the introduction of SARS‐CoV‐2 as a pandemic. COVID‐19 treatment options could be classified as four main classes, including 1) viral entry inhibitors such as monoclonal anti‐SARS‐CoV‐2 antibodies (ex., Bamlanivimab), 2) viral protease inhibitors (ex., lopinavir/ritonavir), 3) viral RNA inhibitors (including remdesivir, favipiravir, and molnupiravir), and 4) Interferons [86, 87].

Vaccination remains a cornerstone for preventing LRTIs caused by pathogens like influenza viruses, COVID‐19 and RSV. For RSV specifically, recent developments include maternal vaccines and monoclonal antibodies such as palivizumab for high‐risk infants. These approaches aim to reduce disease severity by enhancing immunity before exposure to the virus [88, 89].

## 8 Conclusion

**Figure 1:**
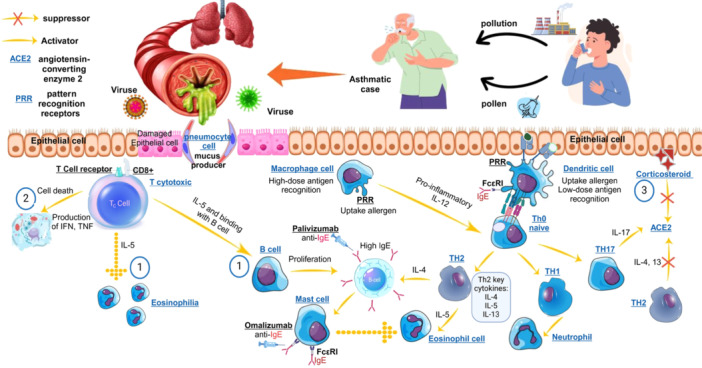
A summary of conclusion about role of viral infections and immune system in asthma is represented. 1‐ In experimental models, T‐cell‐mediated cytotoxicity damages airway cells, and CD8 + T cells release IL‐5, encouraging eosinophilic inflammation and B cell class switching to IgE. 2‐ Virus‐infected CD8 + T cells can enhance interactions with immune cells, increasing Th2‐type responses and allergen‐specific IgE levels. This worsens asthma symptoms and promotes airway inflammation. While CD8 + T cells help clear viruses by inducing apoptosis and producing antiviral cytokines like IFN‐γ and TNF, their involvement in IgE‐mediated responses can negatively affect those with asthma. 3‐ Allergic asthma may reduce COVID‐19 risk by lowering ACE2 expression, which SARS‐CoV‐2 uses to enter cells. Type 2 cytokines like IL‐4 and IL‐13 enhance this effect, while IL‐17 might increase ACE2 levels. Eosinophils can also support antiviral defenses despite being linked to inflammation (this figure was generated by using iStock illustration tool http://www.istockphoto.com).

Viral infections, particularly those caused by human rhinoviruses, are a significant trigger for asthma exacerbations. These infections can lead to heightened serum IgE levels, which play a vital role in the immune response and the worsening of asthma symptoms. Patients with severe allergic asthma, characterized by high IgE levels, are more susceptible to respiratory viruses and often experience more intense virus‐induced asthma exacerbations. The Th2 inflammatory pathway is frequently upregulated during these infections, associated with increased production of cytokines such as IL‐4, IL‐5, and IL‐13, which aggravate asthma symptoms. Additionally, viral infections can compromise the airway epithelium, resulting in greater exposure to allergens and irritants, and disrupt the balance between Th1 and Th2 cytokines, leading to more severe exacerbations. IgE interacts with various immune cells in asthma exacerbations by binding to high‐affinity IgE receptors (FcεRI) on mast cells, basophils, and dendritic cells. This interaction primes these cells for activation upon allergen exposure, releasing proinflammatory mediators that contribute to asthma symptoms. Allergic asthma may protect against COVID‐19 by lowering ACE2 expression, which SARS‐CoV‐2 uses to enter cells. Type 2 cytokines like IL‐4 and IL‐13 enhance this effect, while IL‐17 may increase ACE2 levels, complicating the immune response. Eosinophils can help with antiviral defenses despite being linked to inflammation (Figure 1).

Omalizumab is a monoclonal antibody that lowers IgE levels to prevent allergic responses and asthma exacerbations, particularly in severe allergic asthma patients. Palivizumab is used in infants to prevent RSV infections, which can lead to severe respiratory issues and asthma exacerbations, thereby indirectly aiding asthma management.

## Author Contribution

**Mandana Akhavan:** conceptualization, investigation, visualization, writing – original draft, writing – review and editing. **Parastoo Yousefi:** conceptualization, data curation, writing – original draft, writing – review and editing. **Alireza Tabibzadeh:** conceptualization, data curation, project administration, writing–original draft, writing – review and editing.

## Conflicts of Interest

The authors declare no conflicts of interest.

## AI Usage Declaration

Authors did not use any AI tools for search, drafting, preparing or any other stages of this manuscript.
